# Transient and stable transformation of *Ceratopteris richardii* gametophytes

**DOI:** 10.1186/s13104-015-1193-x

**Published:** 2015-06-04

**Authors:** Linh T Bui, Angela R Cordle, Erin E Irish, Chi-Lien Cheng

**Affiliations:** Donald Danforth Plant Science Center, St. Louis, MO 63132 USA; Department of Biology, University of Iowa, Iowa City, IA 52242 USA

**Keywords:** *Ceratopteris*, Fern, Gametophyte, *Agrobacterium*, Regeneration, Cellulose, GFP, GUS, Hygromycin

## Abstract

**Background:**

Ferns, being vascular yet seedless, present unparalleled opportunities to investigate important questions regarding the evolution and development of land plants. *Ceratopteris richardii,* a diploid, homosporous fern has been advanced as a model fern system; however, the tenuous ability to transform the genome of this fern greatly limited its usefulness as a model organism. Here we report a simple and reliable *Agrobacterium*-mediated method for generating transient and stable transformants of mature *C. richardii* gametophytes.

**Results:**

Transformation success was achieved by enzyme treatment that partially digested the cell walls of mature gametophytes to facilitate *Agrobacteria* infection. Co-incubation of *Agrobacteria* with enzymatically treated gametophytes was sufficient to generate transient transformants at a frequency of nearly 90% under optimal conditions. Stable transformation was achieved at a rate of nearly 3% by regenerating entire gametophytes from single transformed cells from T_0_ gametophytes on selective media.

**Conclusions:**

This transformation method will allow for the immediate observation of phenotypes in the haploid gametophytes of transformed plants, as well as the generation of stably transformed *C. richardii* lines for further analysis. Transformation capability will greatly facilitate gene functional studies in *C. richardii*, more fully realizing the potential of this model fern species. These protocols may be adapted to other plant species that are recalcitrant to *Agrobacterium*-mediated transformation.

**Electronic supplementary material:**

The online version of this article (doi:10.1186/s13104-015-1193-x) contains supplementary material, which is available to authorized users.

## Background

Ferns (*Pteridophyta*) are vascular plants that appeared during evolution before the emergence of seed plants. Compared to other seedless plants such as mosses and liverworts, ferns and fern allies display both ancestral (lack of seeds) and derived (possession of vascular tissue) features, distinguishing them from other clades within the *Plantae*. Extant ferns comprise approximately 12,000 species worldwide and are second only to their sister clade, the angiosperms, in size [1]. Despite the abundance of fern species and the unique opportunity they offer for investigating the evolution of seeds and vascular systems, arguably the two most important events contributing to land plant expansion, ferns are among the most under-investigated land plant clades.

In addition to having a dominant sporophyte generation as in other vascular plants, a salient feature of the ferns is the possession of a free-living, photosynthetic, and macroscopic gametophyte generation that is easily cultured in the laboratory [2]. Therefore, ferns are ideal for investigating such fundamental processes as sporogenesis, gametogenesis, and alternation of generations [3–5]. The diploid homosporous fern, *Ceratopteris richardii,* has been developed into a model fern system. Unlike most homosporous ferns, *C. richardii* lacks a woody rhizome (comparable to the stem in angiosperms) and grows as an annual plant [2]. It has a relatively short life cycle of 120 days from spore to spore under optimum growth conditions and the ability to produce a vast amount of spores [2, 6]. *C. richardii* spores, in the absence of the hormone antheridiogen, will develop into hermaphrodite gametophytes, containing both egg-producing archegonia and sperm-producing antheridia [2, 6]. Under high population density, later-germinating spores develop into smaller male gametophytes, developing antheridia but not archegonia, in response to the antheridiogen produced by earlier-germinated hermaphrodites [6]. Although an array of processes has been investigated using *C. richardii*, such as gametophytic growth and development [7], sex determination [8], the establishment of polarity during spore germination [9, 10], alternation of generations [11–13], and the evolution of vascular cell walls [14], the full potential of this model organism cannot be realized without a simple and reliable genetic transformation system.

RNAi has been used successfully to suppress gene expression in *C. richardii* by biolistic bombardment of DNA constructs expressing double-stranded RNA (dsRNA) against target genes into gametophyte cells. The silencing is systemic, produces visible phenotypes, and can persist in the embryo after fertilization; however, more often than not, the silenced gene tends to re-activate after fertilization [15]. This transient transformation system also has been used successfully in *Pteris vittata* [16]. In another approach, in vitro transcribed dsRNA was directly taken up by germinating *C. richardii* spores, resulting in decreased mRNA levels of target genes when a continual supply of the dsRNA was provided in the medium [17]. Despite a decrease in mRNA level, the dsRNA treatment did not affect the protein level of the target genes, nor did it produce any mutant phenotypes [18]. More recently, a report on stable transformation of the ferns *P. vittata* and *C. thalictroides* spores using biolistic bombardment and *Agrobacterium*-mediated methods, respectively, showed promising results [19]. Unfortunately, because the system lacked a selectable marker for transformed spore cells, it is difficult to determine the transformation efficiency and to evaluate the efficacy of the methods. Another recent report using biolistic bombardment of *C. richardii* callus describes successful stable transformation; however, this method requires the extra step of callus induction from diploid sporophyte explants [20].

Here we report the development of simple, fast, reproducible methods for transiently or stably introducing genes of interest into mature *C. richardii* gametophytes through *Agrobacterium*-mediated transformation. The stable transformation of gametophytes takes advantage of the ease of regenerating hermaphrodites; once a cell is transformed, the antibiotic resistant cell is selected for regeneration. Because gametophytes are haploid, this method permits immediate investigation of the function of gametophytic genes in the regenerated T_0_ hermaphrodites. T_1_ sporophytes are produced simply by allowing the T_0_ hermaphrodites to self-fertilize, or by crossing with other lines.

## Results and discussion

### Partial digestion of the cell wall is critical for *Agrobacterium*-mediated transient transformation of *C. richardii*

We found that the success of an *Agrobacterium*-transformation protocol depends on the choice of tissues for *Agrobacteria* infection. Since both generations of *C. richardii* are free-living and amenable to tissue culture, there are multiple options for the type of tissue, of both gametophytic and sporophytic origins, to be used in transformation. Being haploid, only a single layer of cells, and readily regenerated and propagated on aseptic culture media, the gametophyte presented the ideal choice for *Agrobacterium*-mediated transformation.

**Figure 1:**
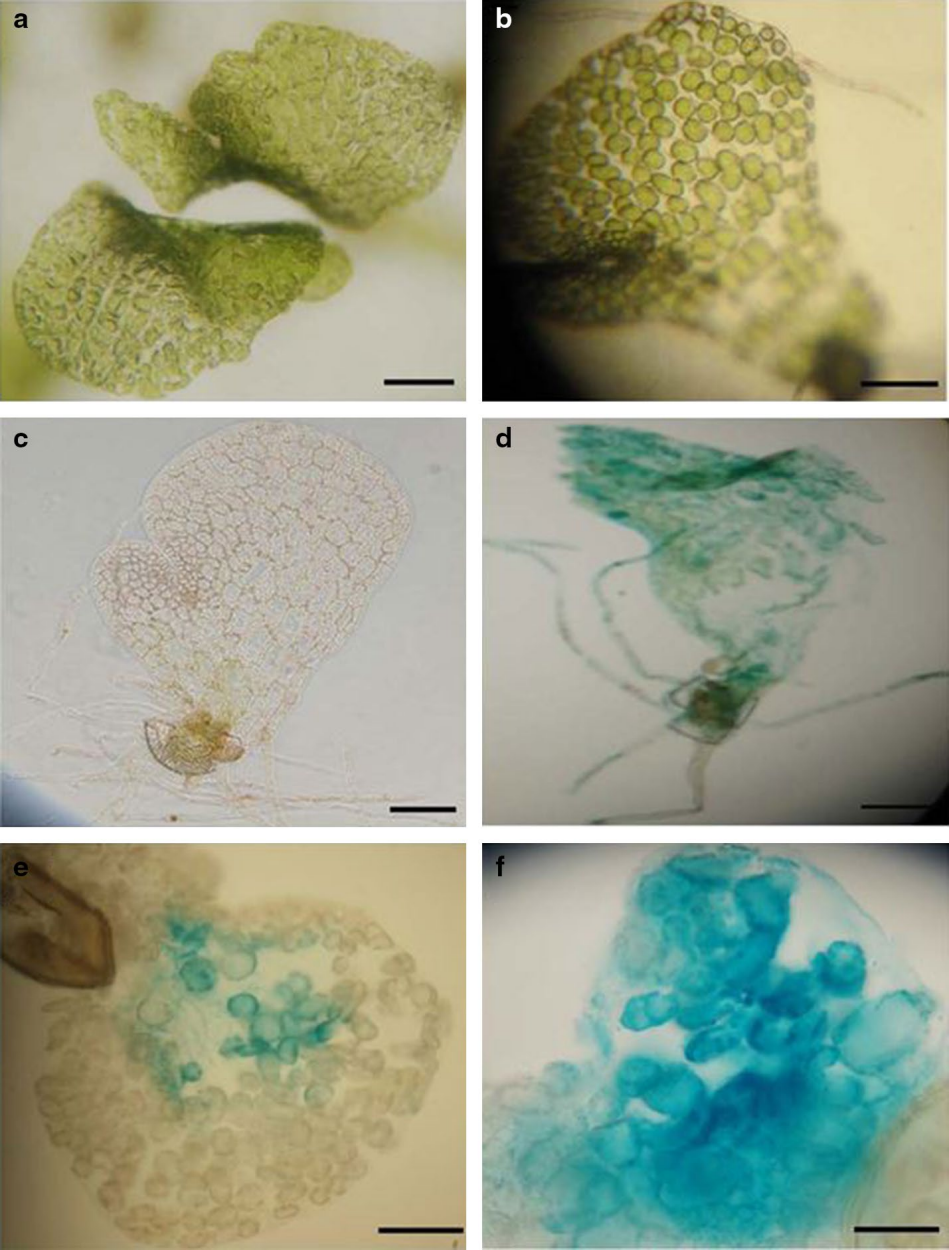
Enzyme treatments facilitate transient transformation. 12-day-old gametophytes treated with 1.5% (w/v) cellulase (**a**) or with 1.5% (w/v) cellulase and 0.5% (w/v) macerozyme (**b**) for 2 h. Histological GUS analysis of transiently transformed gametophytes (**d**–**f**) treated with 1.5% cellulase (w/v) and 0.5% macerozyme (w/v) as in (**b**), or control (no enzyme treatment **c**). *Bar* 0.5 mm.

Spores of the wild type (Rn3) and *hermaphroditic* (*her*) mutant [21] were germinated and grown into mature gametophytes in liquid culture. To facilitate *Agrobacteria* infection, we treated 12-day-old gametophytes with a combination of 1.5% (w/v) cellulase and 0.5% (w/v) macerozyme (containing pectinase, cellulase and hemicellulase). This use of a combination of enzymes is a modification of a previously described protocol for protoplast isolation [9]. After treatment, the gametophytic cells have lost most of their thick cell wall but the prothallus remains largely intact (Figure 1b). The gametophytes were then co-incubated with different *Agrobacterium* strains (GV3101, GV2260 and LBA4404) carrying the vector pMDC139, which contains a *β*-*glucuronidase* (*GUS*) reporter gene [22]. *Agrobacterium* co-incubation was performed for 48 h; the gametophytes were then washed and histologically stained for GUS expression as described in the “Methods”. Nearly 80% of the gametophytes incubated with *Agrobacterium* strain GV3101 stained positive for GUS in most of the cells (Figure 1d–f), whereas the control (without either enzyme treatment or *Agrobacterium* co-incubation) did not stain positive for GUS (Figure 1c). Among the three *Agrobacterium* strains, the percentage of gametophytes showing GUS expression was highest for GV3101, intermediate for GV2260, and lowest for LBA4404 (data not shown). Therefore, the *Agrobacterium* strain GV3101 was chosen for future transformation experiments.

To optimize the conditions for transient transformation in *C. richardii* gametophytes, we tested the effect of different enzyme (cellulase and macerozyme) concentrations and incubation times on transient GUS expression. We found that macerozyme alone has little effect, while cellulase at a concentration higher than 1% (w/v) results in 60% of the samples showing positive GUS staining (Table 1). The optimal condition for transient transformation of *C. richardii* gametophytes using *Agrobacteria* is 1.5% (w/v) cellulase and 0.5% (w/v) macerozyme, since nearly 90% of the gametophytes tested showed GUS signal (Table 1). Of various times tested (1, 2, 3 and 4 h), a 2-h treatment with this enzyme mixture was optimal, keeping the majority of gametophytes both intact and GUS positive (data not shown).

**Table 1:** Effects of enzyme treatment on the success of transient transformation

Enzyme concentration (w/v) with 2 h incubation		Total samples	Number of samples showing GUS signal	GUS positive samples/total
Cellulase	Macerozyme			
0.1%	0	24	1	4.2%
0.5%	0	8	2	25%
1%	0	20	12	60%
1.5%	0	25	14	56%
0.1%	0.25%	25	12	48%
0.5%	0.25%	8	3	37.5%
1%	0.25%	20	9	45%
1.5%	0.25%	8	3	37.5%
0.1%	0.5%	25	17	68%
1%	0.5%	7	3	43%
1.5%	0.5%	15	13	87%
0	0.25%	17	3	17.6%
0	0.5%	12	0	0%
0	0	16	1	6.25%

### Stable transformation of *C. richardii* gametophytes

After successfully establishing a transient transformation protocol for *C. richardii* gametophytes, we applied these treatments to generate stable *Agrobacterium*-mediated transformants. The transformation construct used for stable transformation was pMDC45, which carries a *GFP6* reporter gene and a *hygromycin phosphotransferase* (*HPT*) gene for selection of transgenic plants [22]. Conditions similar to those for transient transformations were used with some modifications to selection and enzyme treatments. After the 48-h co-incubation with *Agrobacteria*, transformed gametophytes were selected on 0.5× Murashige and Skoog (MS) media supplemented with 100 mg L^−1^ cefotaxime and 10 mg L^−1^ hygromycin to kill *Agrobacteria* and to select for the transformants, respectively. This hygromycin concentration was based on the result of a hygromycin sensitivity assay (Additional file 1: Figure S1). However, we discovered that very few of the surviving gametophytes could regenerate after this treatment. The few that did regenerate showed abnormal morphology, and were unable to reproduce or to survive for a prolonged period on this concentration of hygromycin (data not shown). These observations indicated that either the transformation was transient rather than stable, or that the expression of *HPT* gene was not sufficiently high under the control of the *nopaline synthase* (*nos*) promoter. The *nos* promoter is known to be a relatively weak promoter when driving the expression of transgenes in angiosperms [23] and young prothalli of ferns [19].

**Figure 2:**
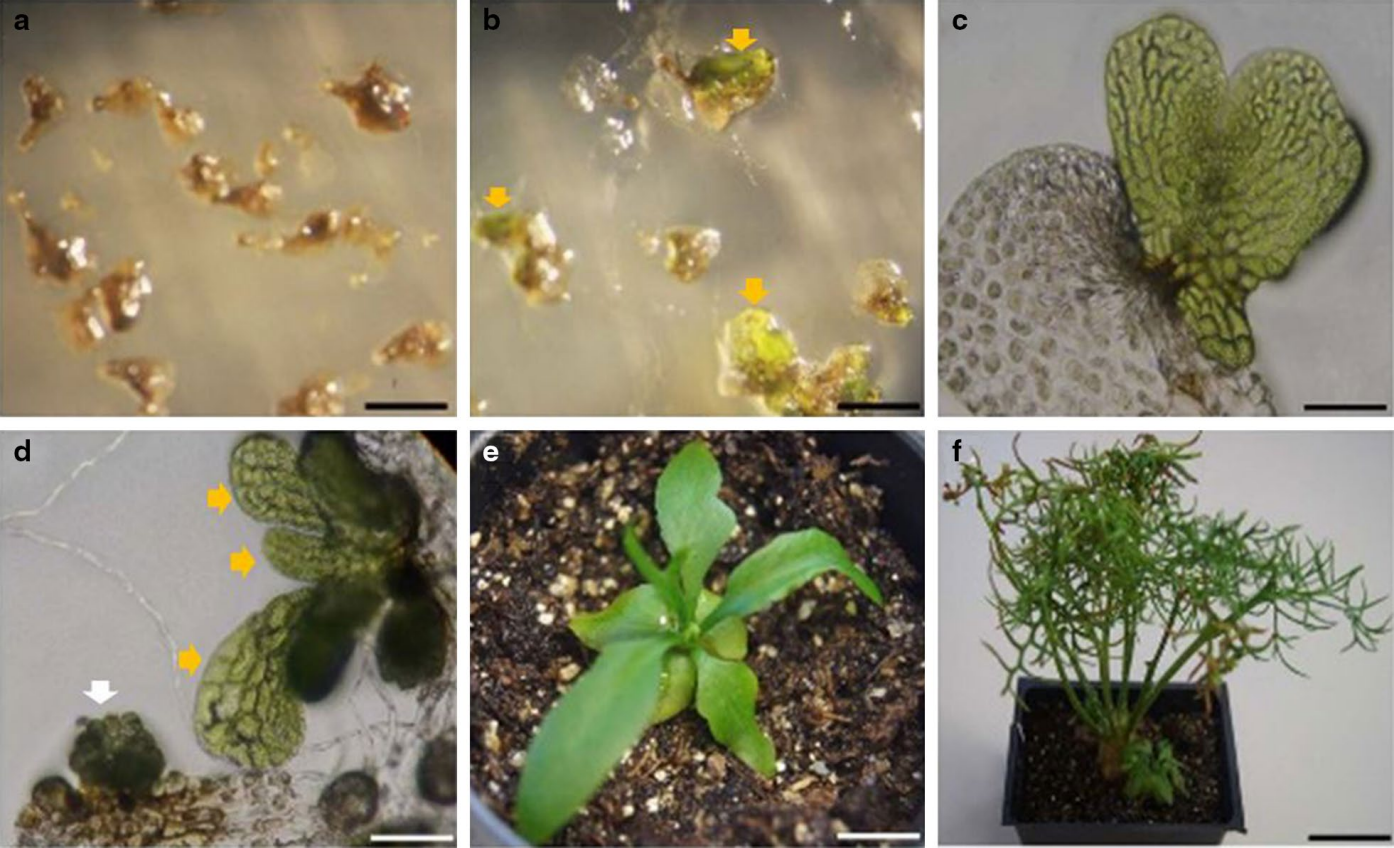
Stable transformation of *C. richardii* gametophytes. 2 weeks after co-incubation with *Agrobacteria*, transformed gametophytic cells remained green and survived on selective media (**b**, *arrows*) while the untransformed gametophytes failed to grow (**a**). A single (**c**), or multiple (**d**, *orange arrows*) regenerated hermaphroditic gametophytes may arise on one gametophyte. A male gametophyte developed from transformed cells of the hermaphroditic gametophytes (**d**, *white arrow*). The young T_1_ sporophytes growing in soil after 1 week (**e**), or 2 months (**f**). All *bars* 1 mm, except in (**c**), *bar* 0.5 mm.

To increase regeneration and survival rate of the transformed gametophytes, we reduced the hygromycin concentration to 2.5 and 5 mg L^−1^. The reduction of hygromycin concentration resulted in more regenerated gametophytes having normal morphology and the ability to reproduce (Figure 2b–d). Gametophytes generated on both concentrations of hygromycin exhibited normal morphology but had a higher regeneration rate on 2.5 mg L^−1^ hygromycin than on 5 mg L^−1^ hygromycin (data not shown). Additionally, the conditions for enzyme treatment before co-incubation with *Agrobacteria* are slightly different between those for stable or for transient transformation. The combination of 1.5% (w/v) cellulase and 0.5% (w/v) macerozyme gave the highest number of gametophytes expressing GUS in transient transformation; however, gametophytes treated with this enzyme combination regenerated poorly on selective media. A 2-h treatment with 1.5% cellulase alone (Figure 1a) prior to *Agrobacterium* co-incubation gave the highest regeneration rate for stable transformation (data not shown). Therefore, a combination of digestion with 1.5% cellulase and selection with 100 mg L^−1^ cefotaxime and 2.5 mg L^−1^ hygromycin was used in experiments described hereon.

On selective media, regenerated gametophytes developed directly from green cells on otherwise dying gametophytes (Figure 2b). Regeneration rarely occurred with a 1:1 stoichiometry, i.e. one regenerated gametophyte from one *Agrobacterium*-incubated gametophyte (Figure 2c), rather, a cluster of multiple regenerated gametophytes developed from one *Agrobacterium*-incubated gametophyte (Figures 2d, 4). Although transformation occurs almost exclusively with hermaphrodites, male gametophytes also regenerated occasionally from transformed cells of the hermaphroditic gametophytes (Figure 2d). It is unclear when the sex was determined as male development is responsive to the hermaphrodite-produced antheridiogen [8] and the males can also convert to hermaphrodites in the absence of antheridiogen and abscisic acid [24]. The regenerated gametophytes of either hermaphrodites or males were indistinguishable from the spore-derived gametophytes and contained functional eggs and sperm that together produced sporophytes (Figure 2e, f).

**Figure 3:**
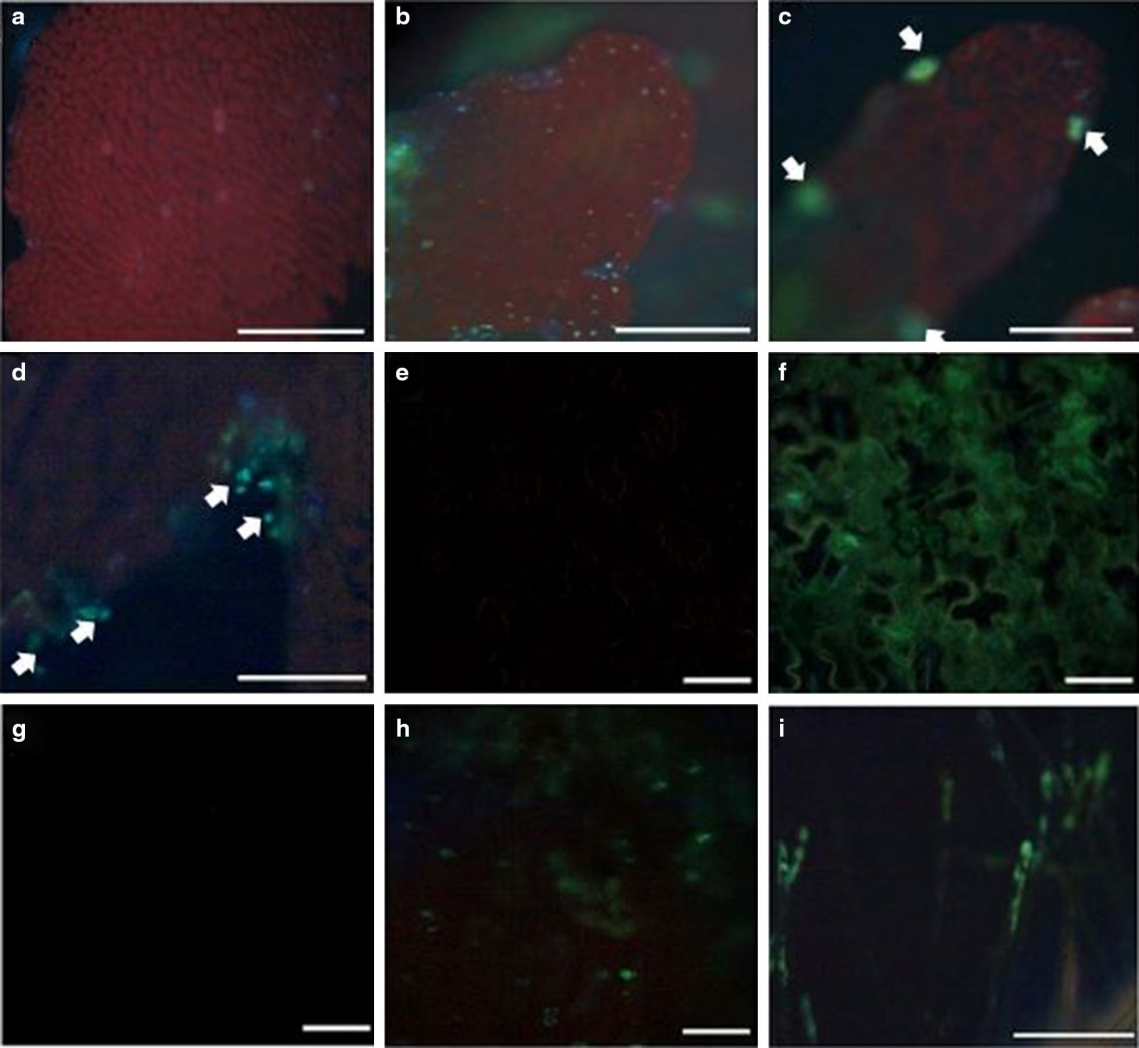
Expression of the reporter gene GFP in transgenic *C. richardii*. GFP signal is seen in both gametophyte (**b**–**d**) and sporophyte (**e**–**i**). In T_0_ gametophyte, GFP signal is present in most of the cells of the prothallus (**b**), and strong GFP signal is seen at the antheridia (**c**, *white arrows*), and in T_1_ gametophytes, strongest in the sperm cells (**d**, *white arrows*) but not in the non-transformed gametophyte (control, **a**). In T_1_ sporophytes, GFP signal is present in all cells of the leaf (**f**), but not the control (**e**). GFP signal is also seen in root tissue (**h**) and root hair (**i**), but not in the root of the non-transformed sporophyte (**g**). *Bars* 1 mm.

The 35S promoter-driven GFP expression was seen in transformed T_0_ gametophytes (Figure 3b, c), T_1_ sporophytes (Figure 3f, h, i), and T_1_ gametophytes (developed from spores produced by T_1_ sporophytes) (Figure 3d), whereas untransformed regenerants did not show any positive signal (Figure 3a, e, g). Overall, GFP was seen in many cells of the transformed gametophytes (Figure 3b), with strongest signal in the antheridia (Figure 3c) due to the presence of GFP in numerous sperm cells, which, lacking chlorophylls, do not autofluoresce. In T_1_ sporophytes, GFP signal was present in all tissues examined, including leaf (Figure 3f), root (Figure 3h) and root hair (Figure 3i), whereas untransformed plants showed no GFP signal in either leaf (Figure 3e) or root tissue (Figure 3g). GFP expression was also observed in the T_1_ gametophytes, with strongest signal in the antheridia and sperm cells (Figure 3d), indicating that the transgene is stably integrated into the genome and inherited by subsequent generations.

### Transformation efficiency and transgene analysis

**Figure 4:**
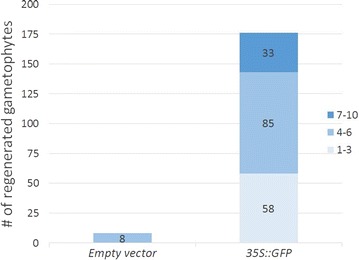
Number of transgenic gametophytes regenerated from the hermaphrodites after co-incubation with *Agrobacteria*. Only eight gametophytes transformed with an empty vector survived selection, compared to 176 survivors transformed with the *35S::GFP* construct. The majority of gametophytes transformed with *35S::GFP* vector (85 gametophytes) gave rise to four to six transgenic gametophytes, 33 of them bore seven to ten gametophytes, and the rest (58 gametophytes) had at least one gametophyte. The total gametophytes used for this analysis were 21,313 and 38,364 for empty vector and *35S::GFP* vector, respectively.

In stable transformation, gametophyte regeneration efficiency was slightly reduced by the enzyme treatment [1.5% (w/v) cellulase for 2 h], with more than 70% of the treated gametophytes regenerating on 0.5× MS media, down from 99% regeneration efficiency in the untreated control (Table 2). Transformation efficiency was calculated using the two following methods. One was based on the number of gametophytes showing regeneration divided by the total gametophytes used in transformation, resulting in an efficiency of 0.5% (Table 3). The other method took into consideration of the ability of a single gametophyte to produce multiple regenerated gametophytes (Figure 4); this calculation was based on the total regenerated gametophytes divided by the total gametophytes used, resulting in a much higher efficiency (1.6–2.6%). This efficiency may be more accurate because the multiple transformed gametophytes regenerated from different parts of a single gametophyte and thus are not likely to be products of the same transformation event. However, the true transformation efficiency is at least four times higher. This is based on the observation that hermaphrodite to male ratio in liquid culture at high density is approximately one to four; males were smaller, most were lost during harvest and the remaining ones do not regenerate.

**Table 2:** The effect of enzyme treatments on regeneration of gametophyte

#	Enzyme treatment	Total gametophytes	Survivors (0.5× MS)	Efficiency
1	0% cellulase	670	663	98.9%
2	1.5% cellulase	692	497	72%

**Table 3:** Transformation efficiency

#	Enzyme treatment [1.5% (w/v) cellulase]	*Agrobacterium* co-incubation (GFP or GUS)	Total gametophytes	Survivors (0.5× MS + 100 mg L^−1^ cefotaxime + 2.5 mg L^−1^ hygromycin)	Efficiency
1	Yes	No	~21,000^a^	8	~0.038%
2	No	No	~21,000^a^	2	~0.009%
3	No	GFP	~21,000^a^	16	~0.076%
5	Yes	GFP	38,364	176	0.459%

^a^Approximate number of gametophytes of three equal portions from one culture.

For transgene analysis, we performed crosses between transformed and wild-type gametophytes that resulted in 64 hygromycin-resistant F_1_ sporophytes. Each T_0_ hermaphroditic gametophyte, representing an independent transformation event, was used either as the maternal or the paternal parent in a cross. Resulting F_1_ sporophytes were analyzed for *HPT* and *GFP6* gene expression. Among the 64 lines examined for GFP, 11 lines expressed GFP in leaf tissue (Additional file 2: Table S1). RT-PCR was performed on total RNA extracted from leaf tissues of 14 transgenic lines (11 *GFP* expressing lines and 3 *GFP* silent lines, Additional file 2: Table S1) using primers specific for the *GFP6* and the *HPT* genes. Among the 14 lines examined, four lines (lines 1, 5, 7 and 13) showed expression of both genes, while all other lines expressed either *GFP6* or *HPT* (Additional file 3: Figure S2), suggesting that one of the transgenes was silenced in the sporophyte or in the next generation. Examination of the expression of these genes in the F_2_ gametophytes should distinguish between these possibilities. Additionally, the different amplicon sizes among the transgenic lines (Additional file 3: Figure S2B) indicates transgene rearrangement of the *GFP6* gene, a phenomenon observed in plant cells transformed with *Agrobacteria* [25].

These F_1_ sporophytes were allowed to produce F_1_ spores, which were used for segregation analysis of hygromycin resistance to further confirm that the T-DNA was integrated into the *C. richardii* genome and to determine copy numbers of the T-DNA. F_1_ spores were germinated on medium containing 20 mg L^−1^ hygromycin to distinguish hygromycin resistant and sensitive spores. Statistical analysis (Table 4) showed that three (lines 1, 2, and 7) out of six lines examined showed a ratio of 1:1 of germinating to dead spores, indicating a single transgene or multiple but tandemly linked transgenes. Only two lines (lines 5 and 13) showed a segregation ratio other than 1:1, indicating multiple insertion of the T-DNA into the genome. Segregation of progeny from line 11 is statistically ambiguous, which may have harbored either single or multiple insertions.

**Table 4:** Spore segregation analysis

Lines	Number of spores		Ratio (germinated:dead)*
	Germinated	Dead	
1	1,149	866	1.3:1^a^
2	3,674	2,760	1.3:1^a^
5	2,062	1,240	1.7:1^b^
7	2,792	2,262	1.2:1^a^
11	3,414	2,334	1.5:1^ab^
13	3,776	1,738	2.2:1^c^
Wildtype	268	3,908	0.07:1^d^

* Chi-square analysis was performed by pairwise comparison between every two lines using the online tool http://www.quantpsy.org/chisq/chisq.htm [26]. Different letters denote the significant difference between conditions at *p* < 0.05 while same letters indicate no significant difference between the two conditions at *p* < 0.05.

Taken together, these results indicate that the transgenes were integrated, in many cases as a single insertion, into the gametophyte genome and the new traits were stably inherited after fertilization and subsequent meiosis, further confirming the efficacy of this stable *Agrobacterium*-mediated transformation method.

## Conclusions

The methods described in this paper allow both transient and stable transformation of *C. richardii*. The transient transformation system allows quick determination of whether and where a promoter acts in the gametophyte generation, among other applications. For stable transformation, the average time required is only 12–15 weeks, thus, the time to harvest T_1_ sporophytes for further analysis is shorter than the 16–18 weeks for the callus bombardment method (not including time needed for callus formation) [20]. This simple and efficient method will greatly facilitate gene functional studies employing overexpression or knock-downs in the fern *C. richardii*. Importantly, by using haploid gametophytes as the starting material, this method is particularly suitable for investigation of gametophytic gene function in the T_0_ hermaphrodites, only days after transformation.

## Methods

### Fern gametophyte cultivation and enzyme treatment

*Ceratopteris richardii* plants used in these experiments were wild-type, genotype Rn3 or *her* mutants, which produce only hermaphrodites, in the *Hnn* background (Carolina Biological Supply, Burlington, NC). Spore germination and gametophyte culture conditions were as follows. Approximately 30 mg of spores were used to inoculate in 75 mL liquid basal medium [0.5× MS salts at pH 6.0] in 250 mL flasks, which were incubated at 28°C under 16-h light/8-h dark cycle. Light was provided by Philips Agro-Lite fluorescent bulbs (Philips Lighting Company, Somerset, NJ, USA) at 90–100 µmol m^−2^ s^−1^. After 3 days, the flasks were moved to a shaker and left shaking at 200 rpm at room temperature for 9 days under the same light conditions. The 12-day-old gametophytes were filtered through a 100 µm nylon mesh filter and treated with a filter-sterilized solution of 0.5 M mannitol plus cellulase or macerozyme, or both enzymes (Plant Phytotechnology). The enzyme treatment was performed in a sterile 100 mm × 20 mm petri dish (Fisher) sealed with Parafilm at 30°C with occasional shaking. Enzyme-treated gametophytes were filtered through a 100 µM nylon mesh, washed several times with 0.5 M mannitol to remove residual enzymes, and then used for transformation.

To examine the effect of enzyme treatment on regeneration, the enzyme-treated gametophytes were similarly washed several times with 0.5 M mannitol to remove enzyme residue, then placed on 0.5× MS vitamin medium at pH 6 without antibiotics for regeneration. The efficiency of enzyme treatment on regeneration was calculated based on gametophyte regeneration 6 weeks after the enzyme treatment.

### Vector construction

The two T-DNA vectors used in this experiment were pMDC139 (*35S::GUS* construct) and pMDC45 (*35S::GFP* construct) [22]. These two vectors were obtained from The Arabidopsis information resource (TAIR). Both vectors were digested with AscI and PacI to remove the *ccdB* gene, self-ligated and transferred into *E. coli* TOP10 strains (Invitrogen). Three-way mating was done with the helper pRK2013 to transfer the T-DNA vectors into *Agrobacterium* strains GV3101, GV2260 and LBA4404.

### *Agrobacterium* preparation and transformation

#### Transient transformation using *Agrobacteria*

The day before transformation, a single colony of *Agrobacteria* carrying the desired vector was grown in 3 mL LB with appropriate antibiotics at 30°C in a rotary shaker set at 120 rpm for 12–16 h. The liquid culture was diluted to OD_600_ of 0.3 in 12 mL YEB medium (without antibiotics), then grown under the same conditions until OD_600_ reached 1.5. Bacteria were harvested by centrifugation at 6,000*g* for 5 min, washed once with 10 mL washing solution (0.5× MS containing 10 mM MgCl_2_ and 100 μM acetosyringone), pelleted by centrifugation at 6,000*g* for 5 min and resuspended in 1 mL washing solution.

*Agrobacterium* suspension was co-incubated with enzyme-treated gametophytes in fern regeneration liquid medium [FRLM (0.5× MS salt media, 10 mM CaCl_2_, 0.375 M mannitol and 0.025 M sucrose) supplemented with 100 μM acetosyringone] at a concentration of OD_600_ of 0.5, in the dark for 48 h. The gametophytes were then washed several times with 0.5 M mannitol and assayed for GUS activity.

#### Stable *Agrobacterium*-mediated transformation and *vir* gene induction

*Agrobacterium* preparation for stable transformation was done as described in [19] with some minor modifications. Briefly, 2 days before the transformation, a single colony of *Agrobacteria* carrying the desired vector was inoculated in 3 mL LB with appropriate antibiotics at 30°C. The culture was grown for approximately 12 h, then 250 µL of the culture was added into 25 mL of the same LB media supplemented with appropriate antibiotics and grown 12–16 h at 28°C with vigorous shaking (120 rpm) in a rotary shaker until OD_600_ reached to 0.5–1.0. Next, 10 mL of the culture was centrifuged at 4,000 rpm for 15 min to collect the cells. Pellets were then resupended in 20 mL of induction medium (IM, as described by Utermark and Karlovsky [27]) supplemented with 200 µM acetosyringone to induce *vir* gene expression. This culture was grown for 24 h at 28°C with low-speed shaking (60 rpm). Enzyme-treated gametophytes in 5 mL of 0.5 M mannitol were co-incubated with 5 mL *Agrobacterium* culture for 15 min at room temperature. Then, the gametophytes were plated on cellophane discs (Research Products International Corp.) overlaid on IM supplemented with 200 µM acetosyringone for 72 h in the incubator with the same conditions as for gametophyte cultivation. Gametophytes were then transferred to 0.5× MS vitamin supplemented with 2% (w/v) sucrose, 0.8% (w/v) agar, 100 mg L^−1^ cefotaxime and appropriate antibiotics for selection. The cellophane overlays were transferred to new, fresh media every 2 weeks. Once they regenerated, the gametophyte clumps were removed from the cellophane discs and transferred onto 0.5× MS salt media supplemented with 0.8% (w/v) agar and appropriate antibiotics to allow the gametophytes to become sexually mature and produce sporophytes.

### Histology

Regenerated gametophytes were photographed using a Canon Powershot A350 mounted onto a compound microscope (Zeiss Axioskop 20) or a dissecting microscope (Leica M60). Histochemical assay for GUS activity was performed according to [28] and [29] with minor modifications. Briefly, the gametophytes were fixed in 80% ice-cold glycerol solution for 15 min. Next, they were vacuum infiltrated for 10 min with GUS staining solution [50 mM sodium phosphate buffer (pH 7.2), 0.5 mM potassium-ferrocyanide, and 1 mM 5-bromo-4-chloro-3-indolyl-β-d-galactopyranoside (X-gal)], then transferred to fresh GUS staining solution and incubated at 37°C for 10 h. To enhance the contrast for GUS staining, the gametophytes were cleared with 70% EtOH to remove chlorophyll before examination with a compound microscope (Zeiss Axioskop 20). For *GFP6* expression analysis, leaf epidermal tissues of both untransformed and transformed sporophytes were peeled to remove the auto fluorescent signal of the chlorophyll. GFP activity was imaged with a Zeiss Axioskop 20 (excitation filter 488 nm, dichroic mirror 510 nm, emission filter 520 nm).

### Transformation efficiency and transgene analysis

Transformation efficiency was calculated from the number of gametophytes surviving on selective media 6 weeks after being transformed, and the total regenerated gametophytes on selective media 12 weeks after that.

Total RNA was extracted from 200 mg sporophyte frond tissue using a modified CTAB protocol [30] followed by a 30-min incubation with DNase (New England Biolabs) to remove DNA. cDNA was synthesized from 500 ng total RNA template using superscript III reverse transcriptase (Life Technologies). Analysis of the transgene expression was done by RT-PCR on the T_1_ sporophytes using the *GFP6* oligos (Fp: GATGTATACGTTGTGGGAGTTGTAG, Rp: CTTGTTGAATTAGATGGTGATGTTAAGG) and *HPT* oligos (Fp: GATGTTGGCGACCTCGTATT, Rp: TAGCGAGAGCCTGACCTATT). Another RT-PCR was performed in parallel with the control UBQ10 oligos (Fp: GATGGCCGTACTCTTGCAGAC, Rp: GGAGACGAAGCACGAGATGA) to ensure the quality of cDNAs used in the experiments. All RT-PCR was performed using the following program: 30 cycles of (95°C for 20 s, 55–58°C for 30 s, 72°C for 30 s).

### Transgene number copy analysis by spore segregation

Crosses were performed as followed: T_0_ transformed hermaphrodites were crossed with wild-type male gametophytes and wild-type hermaphrodites were crossed with sperm collected from T_0_ transformed hermaphrodites. Successful crosses were confirmed by selecting F_1_ sporophytes on media supplemented with 10 mg L^−1^ hygromycin. F_1_ spores were then collected from individual F_1_ sporophytes and germinated on selective media (20 mg L^−1^ hygromycin). Spore segregation analysis was performed by counting the number of germinating spores 21-days after plating and the number was then divided by the number of dead spores to determine the ratios.

## Additional files

Additional file 1:
**Figure S1.** Hygromycin sensitivity assay. Additional file 2:
**Table S1.** Examination of *HPT* and *GFP6* gene expression in heterologous plants resulting from crosses. Additional file 3:
**Figure S2.** Transgene analysis by RT-PCR.

## Abbreviations

dsRNA: double-stranded RNA
*her*: *hermaphroditic*
*GUS*: *β*-*glucuronidase*
*HPT*: *hygromycin phosphotransferase*
MS: Murashige and Skoog
*nos*: *nopaline synthase*
FRLM: fern regeneration liquid medium
