# Supplementary material for: Transient and stable transformation of Ceratopteris richardii gametophytes
Source: BMC Res Notes. 2015 Jun 4;8:214. doi: 10.1186/s13104-015-1193-x (PMC4467839; doi:10.1186/s13104-015-1193-x)
Supplement: Additional file 1: — Figure S1. Hygromycin sensitivity assay. [file 13104_2015_1193_MOESM1_ESM.pdf]

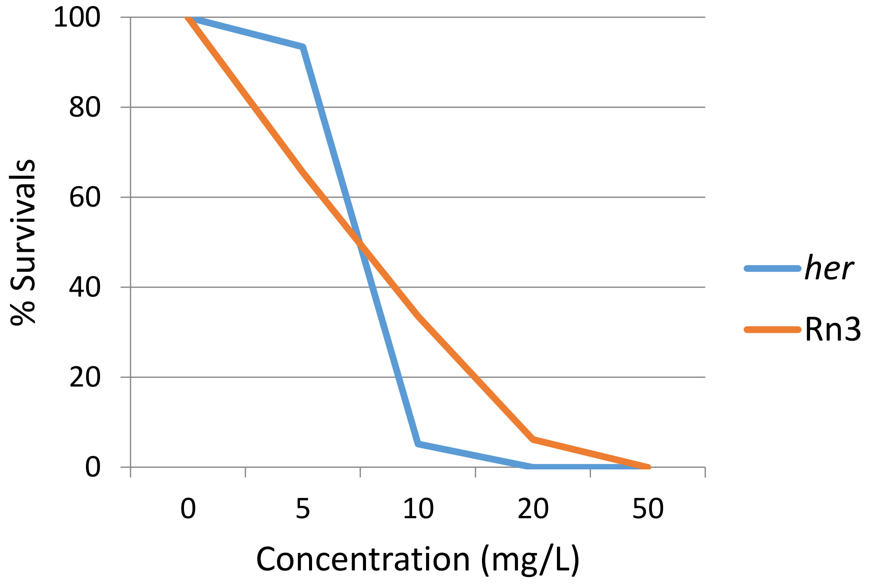

Supplemental Figure 1. Hygromycin sensitivity assay. Effects of hygromycin on gametophyte survival on two *C. richardii* strains, *her* and Rn3. Percentage survival was calculated based on the surviving gametophytes 21d after sowing on 0.5X MS media containing various concentration of hygromycin.
