# Supplementary material for: Transient and stable transformation of Ceratopteris richardii gametophytes
Source: BMC Res Notes. 2015 Jun 4;8:214. doi: 10.1186/s13104-015-1193-x (PMC4467839; doi:10.1186/s13104-015-1193-x)
Supplement: Additional file 2: — Table S1. Examination of HPT and GFP6 gene expression in heterologous plants resulting from crosses. [file 13104_2015_1193_MOESM2_ESM.pdf]

Supplemental Table 1. Examination of *HPT* and *GFP6* gene expression in transgenic plants resulting from crosses

Cross	Line #	Hygromycin resistant[1]	*GFP6* expression[2]
Cross 1: GFP♂ x WT♀	1	+	+
	2	+	-
	3	+	+
	4	+	-
	5	+	+
Cross 2: WT♂ x GFP♀	6	+	+
	7	+	+
	8	+	+
	9	+	+
	10	+	+
	11	+	-
	12	+	+
	13	+	+
	14	+	+

[1]*: hygromycin resistant ability was tested by the ability to survive on 0.5X MS media supplemented with 10mg* L^{-1} *hygromycin.*
[2]*: GFP6 gene expression was examined on epidermal-peeled leaf sample under florescent microscope.*
