# Supplementary material for: Transient and stable transformation of Ceratopteris richardii gametophytes
Source: BMC Res Notes. 2015 Jun 4;8:214. doi: 10.1186/s13104-015-1193-x (PMC4467839; doi:10.1186/s13104-015-1193-x)
Supplement: Additional file 3: — Figure S2. Transgene analysis by RT-PCR. [file 13104_2015_1193_MOESM3_ESM.pdf]

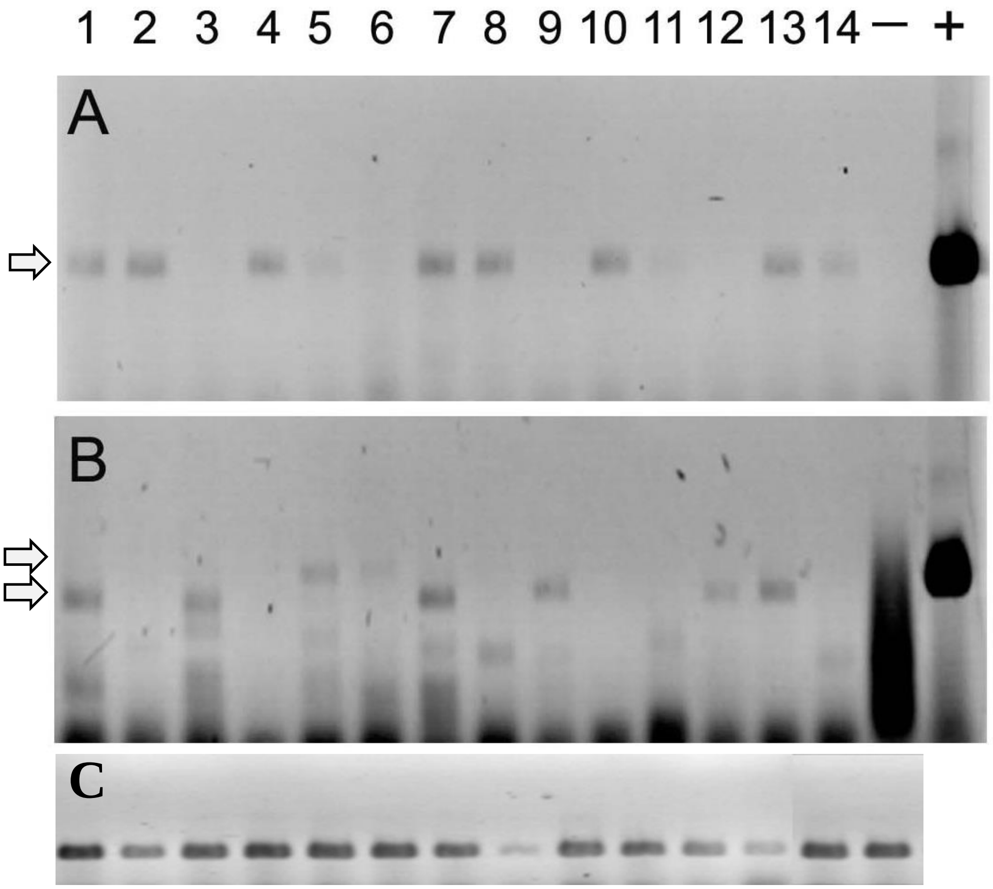

Supplemental Figure 2. Transgene analysis by RT-PCR. *HPT* (A) and *GFP6* (B) expression in the T_1 sporophytes as shown by RT-PCR. Total RNA from independent transgenic T_1 sporophytes (1-14) was used in the experiment; the non-transgenic sporophyte was used as negative control (-) and plasmid containing the transgenes was used as (+) control. (C) RT-PCR with UBQ10 primers was performed in parallel as control. Arrows indicate amplicon size.
